# Porphyrin-Based Fluorescent Probe for Nanomolar Detection of Cu^2+^ and Ni^2+^ Ions

**DOI:** 10.3390/molecules31101739

**Published:** 2026-05-19

**Authors:** So-Hyun Shin, Jihyun Kim, Hyungkyu Moon, T. Sheshashena Reddy, Myung-Seok Choi

**Affiliations:** 1 Department of Materials Science and Engineering, Konkuk University, 120 Neungdong–ro, Gwangjin–gu, Seoul 05029, Republic of Korea; tlsthgus612@konkuk.ac.kr (S.-H.S.); keke1029@konkuk.ac.kr (H.M.); tssreddy@konkuk.ac.kr (T.S.R.); 2 Advanced Materials Program, Department of Materials Science and Engineering, Konkuk University, 120 Neungdong–ro, Gwangjin–gu, Seoul 05209, Republic of Korea; wlguszoq@konkuk.ac.kr

**Keywords:** porphyrin sensor, fluorescence, 2,2′-dipicolylamine, metal cation, copper(II) ion

## Abstract

Copper is an indispensable trace element for maintaining metabolic homeostasis; however, the dysregulation and subsequent accumulation of Cu^2+^ are critically linked to neurodegenerative pathologies, including Alzheimer’s disease in humans. Consequently, the development of robust analytical tools for Cu^2+^ monitoring is of paramount importance. Here, we report a 2,2′-dipicolylamine porphyrin (DPAP)-based fluorescent sensor designed for the precise detection of metal cations. Photophysical investigations reveal that DPAP operates via a rapid turn-off fluorescence mechanism, achieving high-performance sensing in the parts-per-million range. Notably, the probe demonstrates exceptional sensitivity with detection limits of 26.3 nM for Cu^2+^ and 34.8 nM for Ni^2+^. Interference studies demonstrated the selectivity of DPAP for Cu^2+^ over a diverse range of competing metal ions such as Na^+^, Ag^+^, Ni^2+^, Cr^3+^, Pb^2+^, Al^3+^, Fe^2+^, Cd^2+^, and Zn^2+^. These results indicate that DPAP is a sensitive and selective probe suitable for copper ion detection.

## 1. Introduction

Heavy and transition metals are significant environmental pollutants, yet they are essential for the vital functions of many organisms. However, exposure to these metals, whether excessive or deficient, can disrupt metabolic homeostasis or cause environmental toxicity [1,2,3,4]. Due to their inherent toxicity, the precise detection and quantification of these metals are crucial. Fluorescence-based sensors have emerged as highly sensitive and efficient probes for this purpose; consequently, various chemosensors for metal cations have been developed [1,3,5,6,7,8,9,10,11,12,13,14,15,16,17,18,19]. Copper (Cu) is one of the most pervasive transition-metal contaminants. As a key micronutrient and the third most abundant transition metal in the human body [20], approximately 40 μg/L is required for normal metabolism [1,2,8]. Conversely, high concentrations of Cu can induce vomiting, nausea, diarrhea, hepatic or renal damage, and even fatality. Furthermore, Cu is suspected of causing liver damage in infants, and elevated Cu^2+^ levels are implicated in the pathogenesis of Alzheimer’s disease [21]. Therefore, the quantification of trace Cu^2+^ ion is of paramount importance. Highly sensitive fluorescence-based methods have attracted significant attention for the detection of trace amounts of metals [9,20]. Various fluorophores, such as BODIPY [8,22], rhodamine [23,24], coumarin [25], benzimidazole [26,27,28], and naphthalimide [29,30], have been utilized. Among them, porphyrins having a π-conjugated macrocyclic ring and a planar conformation show intense visible light absorption and fluorescence emission. Porphyrins are currently widely applied in photodynamic therapy, organic solar cells, catalysis, and chemosensors [31,32,33,34,35,36,37]. Several research groups reported porphyrin-based Cu^2+^ fluorescent probes [33,34,35,36,37].

In this study, we report the synthesis of a novel fluorescence-based probe, 2,2′-dipicolylamine porphyrin (DPAP), designed for the detection of metal ions, particularly Cu^2+^. This sensor features a porphyrin fluorophore substituted with 2,2′-dipicolylamine (DPA) at the meso position. In the DPAP system, the porphyrin acts as the signaling unit, while the DPA moiety serves as the metal-recognition unit. Notably, the high selectivity of DPA for Cu^2+^ is achieved through direct N–metal interactions.

## 2. Results and Discussion

### 2.1. Synthesis and Characterization

To achieve high-performance fluorescence sensing, the fluorophores must have high quantum efficiency and photostability after metal binding. In addition, to overcome photon attenuation, fluorophores with long emission wavelengths, especially in the near-infrared (NIR) region, are preferred. Porphyrins have been used as fluorophores in fluorescence sensors for many metal ions. Crucially, porphyrin fluorophores have large Stokes shifts between the absorption (approximately 400 nm) and red/NIR emission [33,34,35,36,37]. Furthermore, the DPA ligand forms stable complexes with many metal cations, resulting in high sensitivity [38,39,40,41,42,43,44,45]. We selected DPA because it can efficiently ligate metals in a tridentate binding mode. Further, we assumed that, after coordination with a metal, the lone pair on nitrogen would not be available for conjugation, and fluorescence would be quenched. A previous study showed that DPA, when attached to a TPP moiety, selectively reacted with Cd^2+^ [12].

**Scheme 1:**
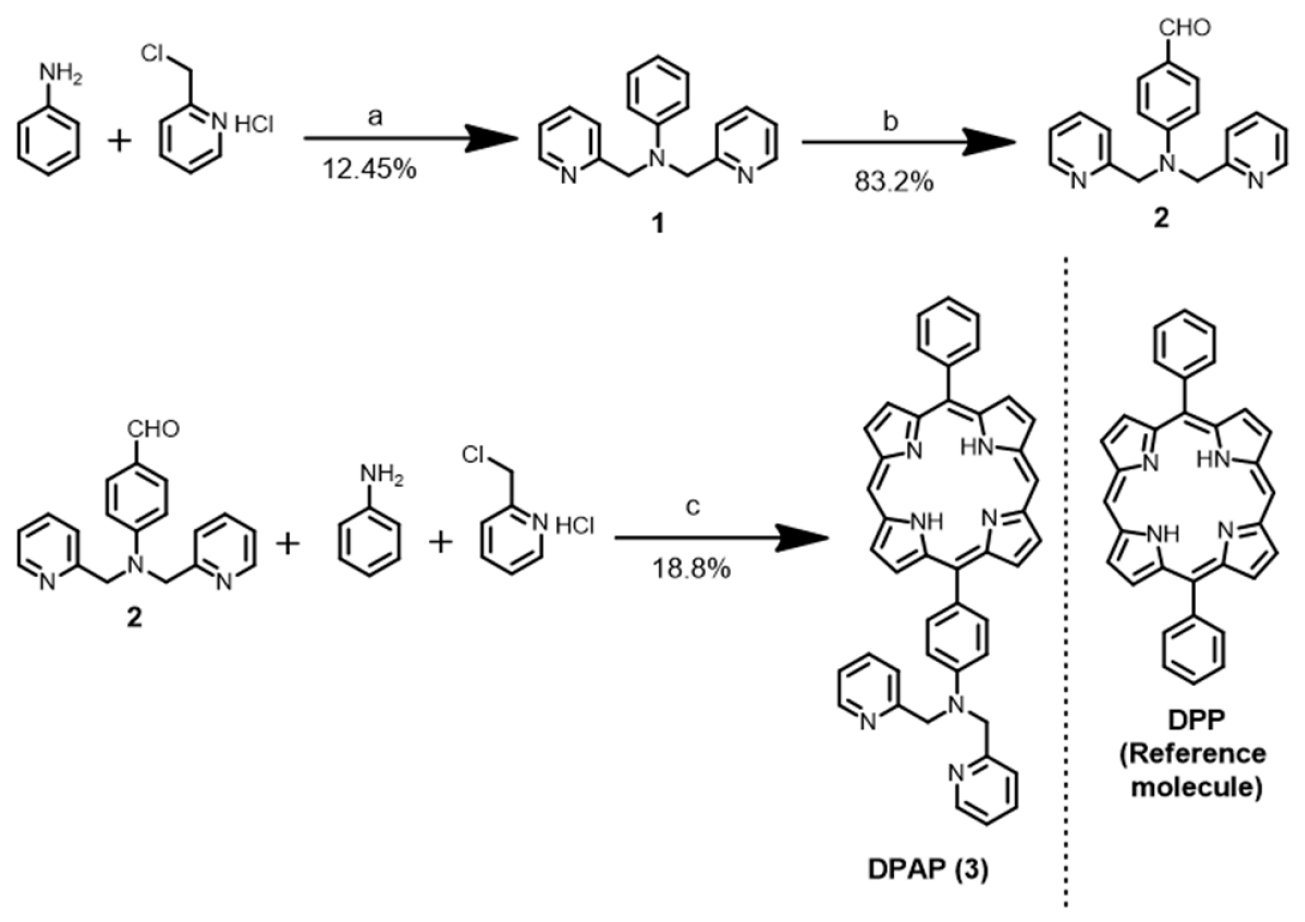
Synthesis of DPAP (**3**). Reaction conditions: (**a**) Hexadecyltrimethylammonium chloride, water, 5 N NaOH/r.t, 24 h; (**b**) POCl_3_, DMF/90 °C, 2 h; (**c**) DCM, TFA/r.t, 6 h; chloranil/1 h; TEA/15 min. Reference molecule DPP.

The synthesis of compounds **1**–**2** is shown in Scheme 1 and follows a literature method [46]. DPAP (**3**) was synthesized by Lindsey cross-coupling condensation, combining two aldehydes and dipyromethane (DPM), followed by oxidation [47]. The synthesis of DPAP was confirmed by MALDI-TOF MS, HRMS, ^1^H-NMR, ^13^C-NMR, and DEPT 135 NMR spectroscopy (Figures S1–S5).

### 2.2. Optical Properties

A stock solution of DPAP was prepared in THF, and all photochemical experiments were carried out at a concentration of 1.5 × 10^−5^ M. Free DPAP produced a prominent absorption band peak maximum at 407 nm (Soret band), attributed to S_0_–S_2_ absorption, and four Q bands at 506, 546, 583, and 639 nm, attributed to S_0_–S_1_ electronic transitions. In addition, DPAP exhibits fluorescence emissions at 647 and 706 nm when excited at 407 nm in THF. The reference molecule diphenyl porphyrin (DPP) exhibited a Soret band at 405 nm and four Q bands at 501, 534, 576 nm, and 633 nm and showed fluorescence emissions at 635 and 700 nm when excited at 405 nm in THF solution.

**Figure 1:**
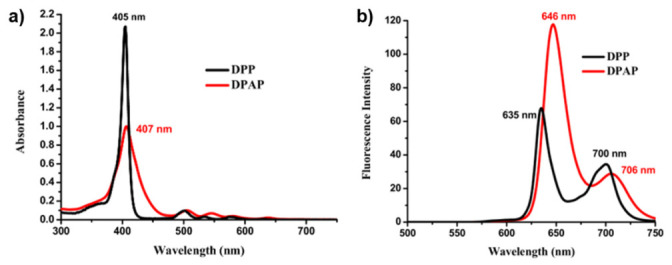
Absorption (**a**) and emission (**b**) (λ_ex_ = 407 nm) spectra of DPP and DPAP in THF (1.5 × 10^−5^ M).

At the same concentration in THF, the absorption spectra of DPP and DPAP differed (Figure 1a): the Soret band of DPAP was broadened, and the peak was red-shifted compared with those of DPP, possibly because of the electron-donating ability of the DPA moiety towards the porphyrin ring.

### 2.3. Effect of Metal Ions on the Absorption and Emission of DPAP

To observe the photophysical response to various metal cations, the UV–vis and fluorescence spectra were measured in the presence of perchlorate salts of Na^+^, Ag^+^, Cu^2+^, Ni^2+^, Cr^3+^, Pb^2+^, Al^3+^, Fe^2+^, Cd^2+^, and Zn^2+^.

As shown in Figure S7a, the Soret band is sharpened, and a hyperchromic shift occurs on metal-ion binding. The spectra for each ion are presented in order of peak intensity. The slight blue shift suggests that M^2+^/DPA coordination decreased the electron-donating ability of DPA, reducing the efficiency of ICT from the DPA subunits to the porphyrin fluorophore. In addition to this electron-withdrawing effect induced by the cations, M^2+^/DPA coordination via the nitrogen might distort the porphyrin ring, decreasing the conjugation of the DPA moiety and fluorophore.

**Figure 2:**
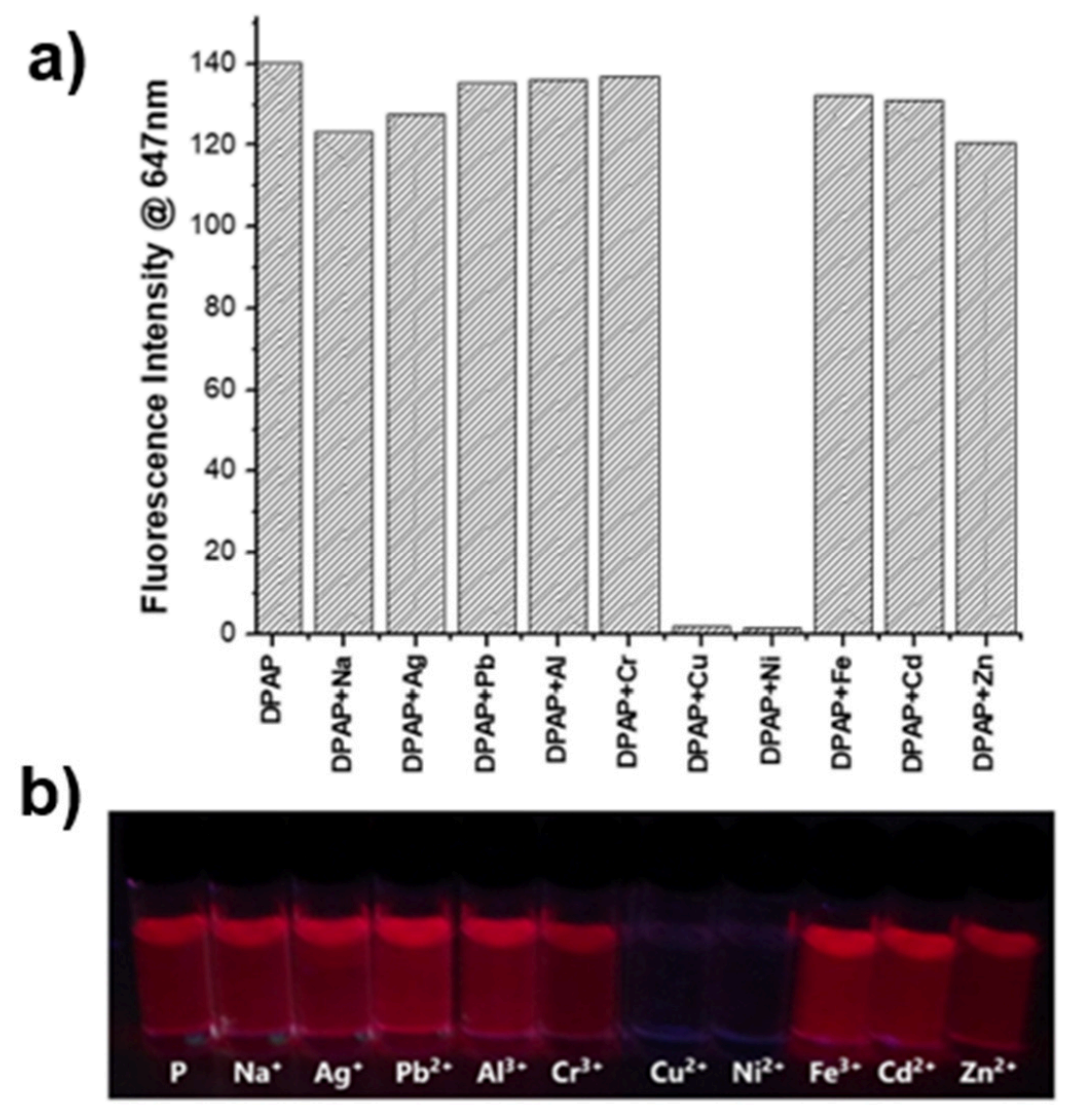
(**a**) Fluorescence emission response of DPAP (1.5 × 10^−5^ M) in the presence of various metal ions (10 eq) and (**b**) images under UV light (*λ*_em_ = 365 nm) of DPAP (1.5 × 10^−5^ M) in the presence of various metal ions (1.5 × 10^−4^ M, 10 eq).

Next, the emission spectra of the sensors were measured at an excitation wavelength of 407 nm. Figure 2 and Figure S7b shows that the fluorescence intensity is reduced by the coupling of the porphyrin sensor with various metals. The spectra for each ion are presented in order of peak intensity. The metal ions are chelated by the DPA functional group, a process that depends on the ion size and binding affinity.

In Figure S7a, there remain four distinct Q bands, indicating that the metal ions do not bind to the porphyrin ring, and this is consistent with their high affinity for DPP. As shown in Figure S6, the UV–vis and fluorescence spectra of DPP showed no changes in the presence of metal cations, indicating that the DPA groups are the binding sites.

In the case of fluorescence, a slight metal-ion-dependent change was observed; however, a significant quenching was observed in the presence of Cu^2+^ and Ni^2+^. This is because the bite angle of DPAP is commensurate with the size of these metal ions. Figure 2b shows images of DPAP under UV light (λ_em_ = 365 nm), revealing considerable fluorescence quenching in the presence of Cu^2+^ and Ni^2+^. Therefore, we focused on the use of the DPAP-based sensor for Cu^2+^ and Ni^2+^ detection. The reference molecule DPP fluorescence is not affected by various metal ions, indicating that there is no interaction/binding between DPP and metal ions (Figure S6).

The fluorescence quantum yields of DPAP after the addition of Cu^2+^ and Ni^2+^ were calculated using the reference standard TPP (QY = 0.11). The quantum yield of DPAP before the addition of metal cations was 0.1018, and after the addition of Cu^2+^ and Ni^2+^, the corresponding quantum yields of DPAP-Cu^2+^ and DPAP-Ni^2+^ were 0.0016 and 0.0033, respectively. Further, the fluorescence quenching percentages of DPAP after the addition of Cu^2+^ and Ni^2+^ were 99.1% and 98.5%, respectively. In terms of quenching and quantum yield changes, DPAP exhibited the highest reactivity towards Cu and Ni ions. Due to the paramagnetic nature of the Cu^2+^ ion upon binding with the DPAP, the DPAP fluorescence is quenched [48]. Furthermore, we investigated the quenching process mechanism using TCSPC fluorescence lifetime data. DPAP showed 8.65 ns, and DPAP+Cu^2+^ ion showed 8.31 ns fluorescence lifetimes, respectively. These fluorescence lifetime results indicate that fluorescence quenching may occur through static quenching [33].

**Figure 3:**
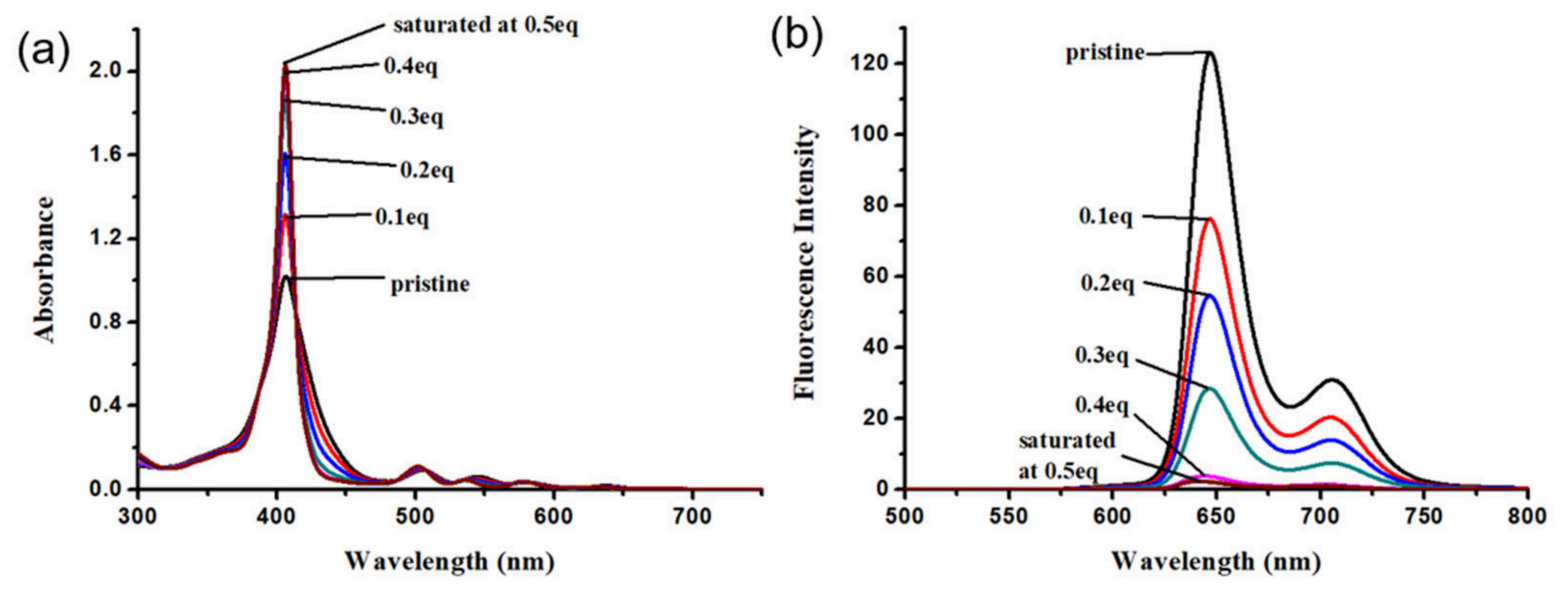
Absorption (**a**) and emission (**b**) (λ_ex_ = 407 nm) spectra of DPAP (1.5 × 10^−5^ M) with an increase in Cu^2+^ concentration (1 eq = 1.5 × 10^−5^ M).

The titration of DPAP with increasing concentrations of Cu^2+^ led to a decrease in emission intensity, as shown in Figure 3. No further changes were observed beyond the addition of 0.5 eq. The titration of DPAP with Ni^2+^ led to fluorescence quenching (Figure S4).

The LOD of DPAP for the two metal ions in THF was calculated from a linear fit of the maximum emission intensity at 647 nm and the quencher concentration (Figures S5 and S6). The calculated LODs are 26.3 and 34.8 nM (≈2.0 ppb, *R*^2^ = 0.98), respectively, for Cu^2+^ and Ni^2+^ based on Equation (1). The previously reported porphyrin based Cu^2+^ ion sensors details were given in Table S1 (sensing type, solvent conditions, detection limits, and response time). Porphyrin 1 and porphyrin 5 are showing turn-off sensing behavior, as does DPAP, but in selectivity and sensitivity terms, they are superior to DPAP. DPAP shows superiority over porphyrin 2, porphyrin 3, and porphyrin 5 with respect to detection limit and response time (Table S1) [33,34,35,36,37].

### 2.4. Binding Stoichiometry and Binding Affinity

To understand the binding behavior and determine the stoichiometry of the DPAP-Cu^2+^ complex, Job’s plot analysis was carried out [6] (Figures S7 and S8). The changes in the emission intensity (*I*_0_ − *I*) at 647 nm versus the mole fraction of Cu^2+^ were measured while maintaining the concentrations of Cu^2+^ and DPAP at 1.5 × 10^−5^ M. The maximum change in emission intensity was observed at a molar fraction of approximately 0.5–0.6. In addition, in the case of Ni^2+^, the results show that the DPA-based sensor for Ni^2+^ metal ion forms 1:1 metal–ligand complexes.

**Figure 4:**
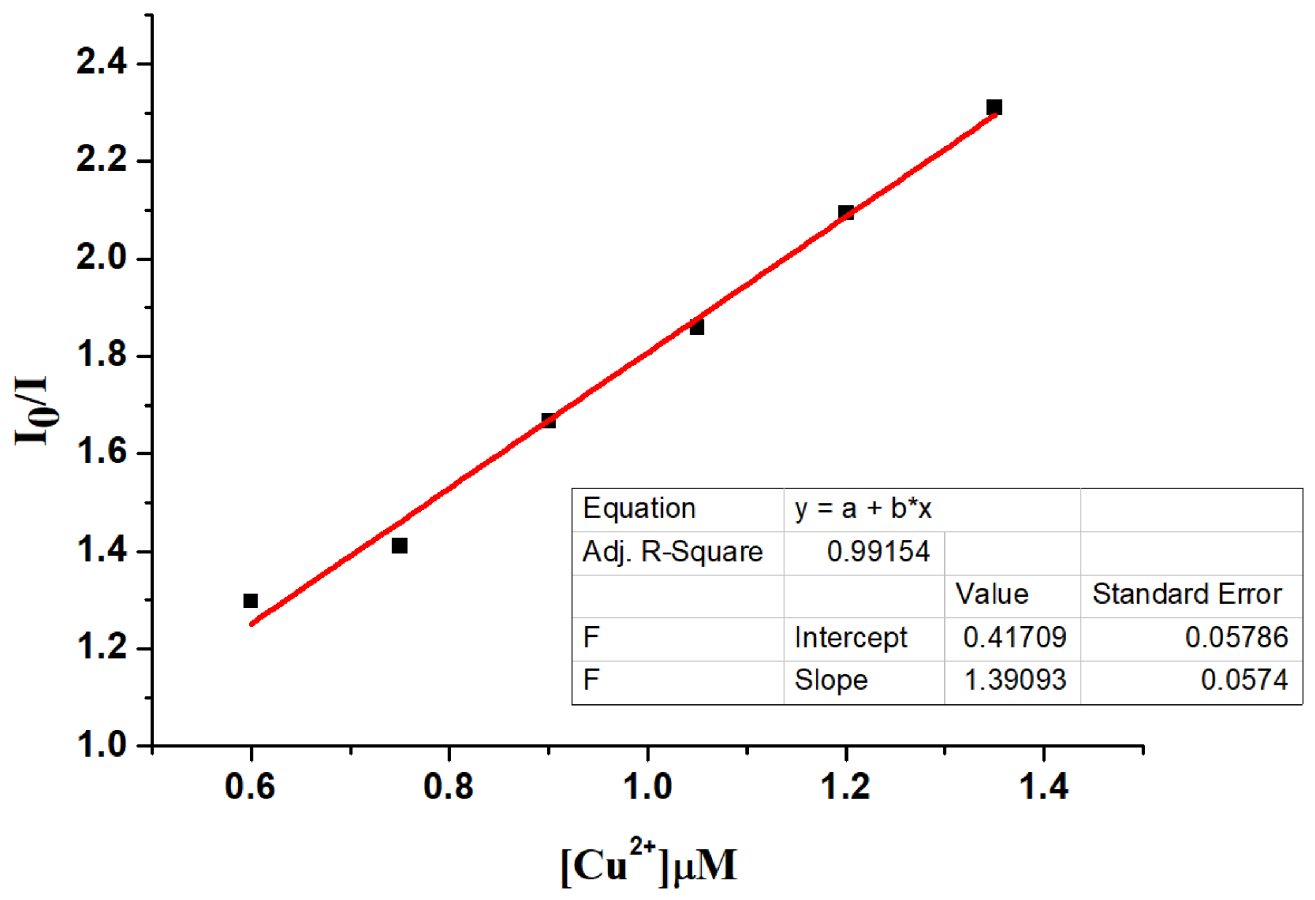
Stern–Volmer plot of the quenching emission at 647 nm on the interaction of DPAP with Cu^2+^ (*K*_sv_ = 1.39 × 10^6^ M^−1^).

As shown in Figure 4 and Figure S9, near-linear Stern–Volmer plots were obtained at low quencher (metal ion) concentrations; these plots were used to investigate the “kinetics” of the quencher binding affinity. Linear fitting yielded a coefficient of determination (*R*^2^) of >0.99, and the obtained Stern–Volmer constants were 1.39 × 10^6^ and 7.71 × 10^5^ M^−1^, respectively, for Cu^2+^ and Ni^2+^, indicating an increase in binding affinity in that order.

**Figure 5:**
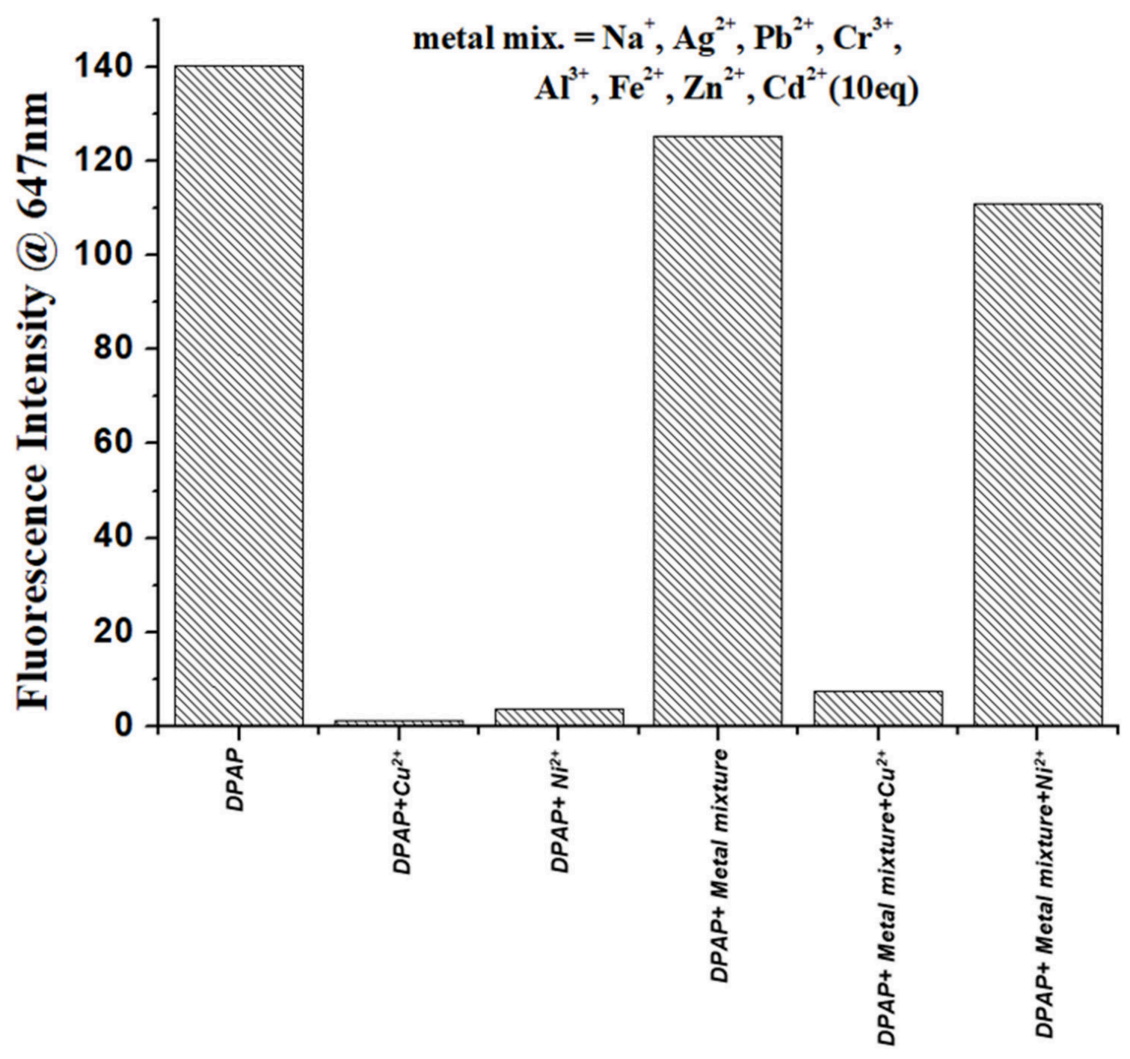
Fluorescence emission response of DPAP (1.5 × 10^−5^ M) in the presence of Cu^2+^, and Ni^2+^ (1 eq) and interference effects in the presence of a metal mixture (10 eq).

Next, interference (matrix) effects were investigated using metal cations known to bind to the DPA moiety, such as Zn ions [12,13,39,40,41,42,43,44,45,46]. A comparative study was carried out by measuring a low-concentration (1 eq) mixture of Cu^2+^ and Ni^2+^ and a high-concentration (10 eq) mixture of interfering metals. As shown in Figure 5, Cu^2+^ completely quenched the fluorescence, unlike the other metals, and no interference was observed. Although Ni^2+^ quenched the fluorescence, interference was observed in the presence of the metal mixture, and the fluorescence intensity increased again because of its low binding affinity. These results clearly demonstrate the high specificity of our sensor for Cu^2+^, even in a complex matrix of metal ions.

To investigate the interference effect of each metal ion and metal ion mixtures on the detection ability of DPAP for Cu^2+^ and Ni^2+^, the fluorescence response was measured in the presence of other metal ions (Figure 5 and Figures S10 and S11). Exposure of DPAP to a mixed metal-ion solution containing Na^+^, Ag^2+^, Pb^2+^, Cr^3+^, Al^3+^, Fe^2+^, Zn^2+^, and Cd^2+^ (10 equivalents each) does not significantly alter the fluorescence intensity, which remains comparable to that of free DPAP. In contrast, the addition of Cu^2+^ to the mixed-metal system quenched the fluorescence of DPAP, highlighting the high selectivity of DPAP for Cu^2+^ even under competitive conditions. The addition of Ni^2+^ to the mixed-metal system resulted in partial quenching. In addition, DPAP has greater selectivity for Cu^2+^ than for Ni^2+^ ions, as shown in Figures S10 and S11. Taken together, these results highlight the Cu^2+^selective fluorescence quenching exhibited by DPAP, underscoring its potential utility as a highly selective fluorescent probe for Cu^2+^ detection.

**Figure 6:**
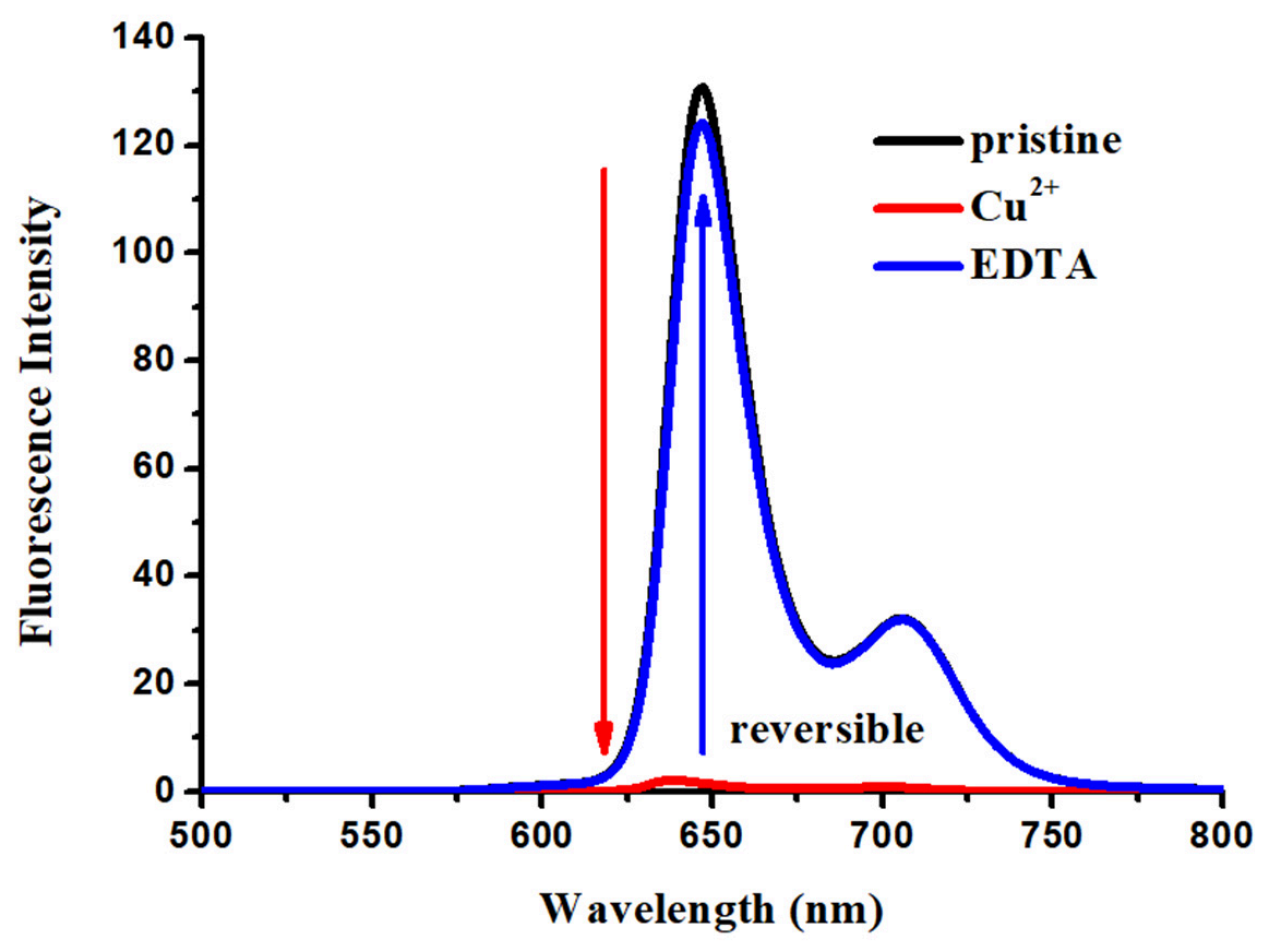
Emission spectra (λ_ex_ = 407 nm) of the DPAP-Cu^2+^ complex (1.5 × 10^−5^ M) in the presence of EDTA.

Moreover, the optical response of DPAP to Cu^2+^ is fully reversible. In addition, when ethylenediaminetetraacetic acid (EDTA) was added to the complex solution, the fluorescence signal at 647 nm completely recovered (Figure 6) [14].

## 3. Experimental Section

### 3.1. Materials

All chemicals for synthesis were purchased from commercial suppliers and used as received. Aniline, phosphorus(V) oxychloride, and all metal salts were purchased from Sigma–Aldrich, Republic of Korea. Hexadecyltrimethylammonium chloride, benzaldehyde, dipyromethane (DPM), and chloranil were purchased from Tokyo chemical industry company limited, Japan. Picolyl chloride hydrochloride was purchased from Acros Organics, Republic of Korea. *N*, *N*′-Dimethylformamide (DMF) and dichloromethane (DCM) were purchased from Samchun Company, Republic of Korea. Trifluoroacetic acid (TFA) was purchased from Daejung Corporation, Republic of Korea. Triethylamine (TEA) was purchased from Duksan Corporation, Republic of Korea.

### 3.2. Measurements

^1^H-NMR spectra were recorded at 600 MHz and are reported using the standard abbreviations: s: singlet, d: doublet, t: triplet, m: multiplet, and the coupling constants, *J*, are given in hertz. The chemical shifts are reported in parts-per-million (ppm), using tetramethylsilane (0 ppm) and CDCl_3_ as standards. ESI mass spectra were recorded using a Thermo Scientific/Q exactive high-resolution orbitrap mass spectrometer, Germany. UV−vis absorption and fluorescence emission spectra were recorded using a Shimadzu UV-2450 spectrometer, Japan and a Hitachi F-7000 fluorescence spectrophotometer, Japan respectively. The slit size for excitation and emission was 5 mm. The emission spectra were measured with excitation at 407 nm. All spectra were recorded at room temperature (r.t.) in a quartz cuvette having a path length of 10 mm.

A stock solution of DPAP was prepared in tetrahydrofuran (THF) at a concentration of 1.5 × 10^−5^ M for excitation and emission measurements and stored in a cold, dark place before use. To test the sensing performance and interference effects, various metal-ion solutions (Na^+^, Ag^+^, Cu^2+^, Ni^2+^, Cr^3+^, Pb^2+^, Al^3+^, Fe^2+^, Cd^2+,^ and Zn^2+^ as perchlorate salts) were prepared in water. Column chromatography was performed on Merck silica gel (230–400 mesh).

### 3.3. DPAP Synthesis

Compounds **1** and **2** were synthesized following procedures reported in the literature [46]. Compound **2** (DPA aldehyde) (0.519 g, 1.712 mmol), benzaldehyde (0.18 mg, 1.712 mmol), and DPM (0.5 g, 3.424 mmol) were dissolved in dichloromethane (DCM; 600 mL), and the mixture was degassed for 10 min by sparging the solution with N_2_. After the addition of TFA (0.234 g, 0.6 eq), the resulting solution was stirred at room temperature for 6 h in the dark. To this reaction mixture, chloranil (1.05 g, 4.28 mmol) was added, and the reaction mixture was stirred for another 1 h at r.t. After reaction completion, the mixture was neutralized with TEA (4 mL), filtered through a Büchner funnel filled with silica gel, and washed with chloroform, completing the work-up process. The crude compound **3** (i.e., DPAP) was purified by silica-gel column chromatography using chloroform (0.5% *v*/*v* methanol) as the eluent, yielding a magenta solid in 18.8% yield. ^1^H-NMR (600 MHz, chloroform-*d*) *δ* 10.28 (s, 2H, meso), 9.37 (d, 4H, *J* = 4.68 Hz, β), 9.18 (d, 2H, *J* = 4.40 Hz, β), 9.06 (d, 2H, *J* = 4.68 Hz, β), 8.70 (d, 2H, *J* = 4.40 Hz, aromatic), 8.27−8.26 (m, 2H, aromatic), 8.08 (d, 2H, *J* = 8.53 Hz, aromatic), 7.84−7.79 (m, 5H, aromatic), 7.60 (d, 2H, *J* = 7.70 Hz, aromatic), 7.30–7.29 (m, 2H, aromatic), 7.16 (d, 2H, *J* = 8.53 Hz, aromatic), 5.13 (s, 4H, aliphatic), and -3.05 (s, 2H, NH). ^13^C-NMR (125 MHz, chloroform-*d*) δ 158.9, 149.9, 147.7, 147.3, 145.1, 141.5, 137.3, 136.3, 134.9, 131.7, 131.4, 130.9, 127.7, 122.4, 121.3, 118.7, 118.2, 116.8, 112.1, 111.4, 105.2, 57.6. MS (MALDI-TOF-MS): *m*/*z* was calculated for C_44_H_33_N_7_ [M+ H]^+^: 660.283; found: 660.471. HRMS (ESI-TOF): *m*/*z* calculated for C_44_H_33_N_7_ 660.2870 [M+ H]^+^, measured 660.2862 [M+ H]^+^.

### 3.4. Limit of Detection

The limit of detection (LOD) was calculated using fluorescence titration with Equation (1).

LOD = 3*σ*/*k* (1)

Here, *σ* is the standard deviation of the emission intensity of DPAP, and *k* is the slope of the emission intensity plotted against the concentration. To determine the signal-to-noise ratio, the emission intensity of pristine DPAP without metal ions was measured four times, and the standard deviation of the blank measurements was determined to be 0.221736. Three independent duplicate measurements of the emission intensity were performed in the presence of Ni^2+^ and Cu^2+^, and the average value of each intensity was plotted as a function of cation concentration to determine the slope.

### 3.5. Quantum Yield Measurements

The quantum yield was calculated using Equation (2).

(2) $$\mathrm{QY}=\frac{I}{I_{R}}\times \frac{A_{R}}{A}\times \frac{\eta^{2}}{\eta_{R}^{2}}\times QY_{R}$$

Here, *QY* and *QY_R_* are the quantum yields of the sample and reference, respectively, *A* and *A_R_* are the absorbances of the sample and reference, respectively, and *I* and *I_R_* are the areas of the emission peaks for the sample and reference, respectively. Tetraphenyl porphyrin (TPP), which has a quantum yield of 0.11, was used as a reference.

### 3.6. Binding Constants and Stoichiometry

The binding (*K_sv_*) and quenching constants for the interaction of the metal cations with the probe were determined using Stern–Volmer plots, Equation (3) [49,50].

(3) $$\frac{I_{0}}{I}=1+K_{sv}{\left[Q\right]}_{q}$$

Here, *I*_0_ is the initial emission intensity of DPAP before the addition of the quencher, *I* is the emission intensity at a given concentration of quencher Q ([Q]), and *K*_sv_ is the Stern–Volmer constant.

In addition, Job’s plots were constructed to determine the stoichiometry of the complexation process between the metal ions and dye ligand [6,9,51]. In the plots, the *x*-axis represents the mole fraction, which is the ratio of the cation concentration to the total concentration of DPAP and cations. The total molar concentrations of the probe and metal ion were constant (1.5 × 10^−5^ M), and the difference in the emission intensity (*I*_0_ − *I*) between DPAP and the DPAP–M^2+^ complex at 647 nm versus the mole fraction was measured.

## 4. Conclusions

A novel fluorescence-based sensor, DPAP, was successfully synthesized by coupling a DPA metal-recognition moiety to a porphyrin fluorophore for the detection of transition metals, with a primary focus on Cu^2+^. The sensor shows ‘turn-off’ fluorescence, where the addition of less than 1.0 molar equivalent amount of Cu^2+^ ions was sufficient to induce complete fluorescence quenching. The sensor achieved a limit of detection (LOD) of approximately 2.0 ppb for both Cu^2+^ and Ni^2+^, demonstrating its potential for trace-level monitoring. Our results from quantum yield and Stern–Volmer analyses revealed the quenching efficiency of Ni^2+^ < Cu^2+^, suggesting the high affinity of the DPAP probe for Cu^2+^ ions. Interestingly, DPAP exhibited selectivity for Cu^2+^ even in a mixture of metal ions.

## Data Availability

All data and materials described in this work are available in this article or in the Supplementary Materials.

## Supplementary Materials

The following supporting information can be downloaded at: https://www.mdpi.com/article/10.3390/molecules31101739/s1. Figure S1: MALDI-TOF MS of DPAP; Figure S2: ESIMS spectra of DPAP; Figure S3: ^1^H-NMR spectrum of DPAP; Figure S4: ^13^C-NMR spectrum of DPAP; Figure S5: DEPT135 NMR spectrum of DPAP; Figure S6: Absorption and emission spectra of DPP in the presence of various metal ions; Figures S7 Absorption and emission spectra of DPAP in the presence of various metal ions; Figures S8: Absorption and emission spectra of DPAP titration in the presence of Ni^2+^ ion; Figures S9 and S10: LODs of DPAP; Figures S11 and S12: Job’s plots of DPAP; Figure S13: Stern–Volmer plot of DPAP; Figures S14–S16: Interference effects of different metal ions (10 eq); Table S1: Previously reported porphyrin molecules for the detection of Cu^2+^ ions with sensors type solvent, LOD and response time.
