# Supplementary material for: Porphyrin-Based Fluorescent Probe for Nanomolar Detection of Cu2+ and Ni2+ Ions
Source: Molecules. 2026 May 19;31(10):1739. doi: 10.3390/molecules31101739 (PMC13209248; doi:10.3390/molecules31101739)
Supplement: Supplementary file 1 [file molecules-31-01739-s001.zip › molecules-4272231-supplementary.pdf]

# Supporting Information

## Porphyrin-Based Fluorescent Probe for Nanomolar Detection of $Cu^{2+}$ and $Ni^{2+}$ Ions

So-Hyun Shin[1], Jihyun Kim[2], Hyungkyu Moon[1], T. Sheshashena Reddy[1], and Myung-Seok Choi[2]*

[1] *Department of Materials Science and Engineering, Konkuk University, 120 Neungdong–ro, Gwangjin–gu, Seoul, South Korea.*

[2] *Advanced Materials Program, Department of Materials Science and Engineering, Konkuk University, 120 Neungdong–ro, Gwangjin–gu, Seoul, South Korea.*

*Corresponding author. *E–mail address*: mchoi@konkuk.ac.kr (Myung–Seok Choi)

**Contents**

**Figure S1.** MALDI-TOF MS of DPAP
**Figure S2.** ESIMS of DPAP
**Figure S3.** $^{1}H$-NMR spectrum of DPAP.
**Figure S4**. $^{13}C$ NMR spectrum of DPAP.
**Figure S5**. DEPT135 spectrum of DPAP
**Figure S6.** Absorption and emission spectra of DPP in the presence of various metal ions.
**Figure S7.** Absorption and emission spectra of DPAP in the presence of various metal ions.
**Figures S8.** Absorption and emission spectra of DPAP titration in the presence of $Ni^{2+}$ ion.
**Figures S9–S10.** LODs of DPAP.
**Figures S11–S12**. Job's plots of DPAP.
**Figures S13.** Stern–Volmer plot of DPAP.
**Figures S14 and S15.** Interference effects of different metal ions (10 eq).
**Table 1.** Previously reported porphyrin molecules for the detection of $Cu^{2+}$ ions with sensor type, solvent, LOD and response time.

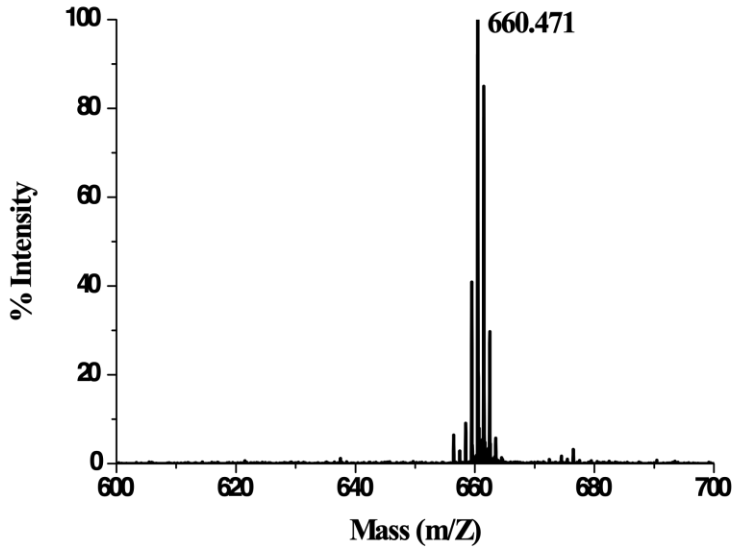


**Figure S1.** MALDI-TOF MS of DPAP.

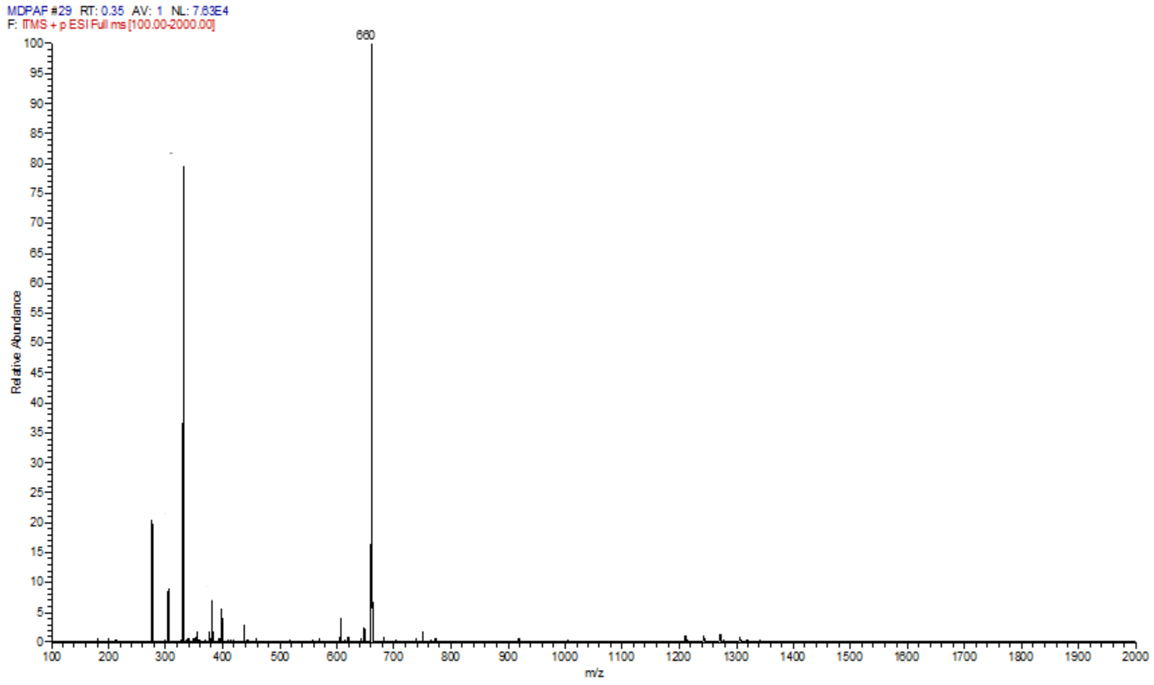


**Figure S2.** ESIMS of DPAP

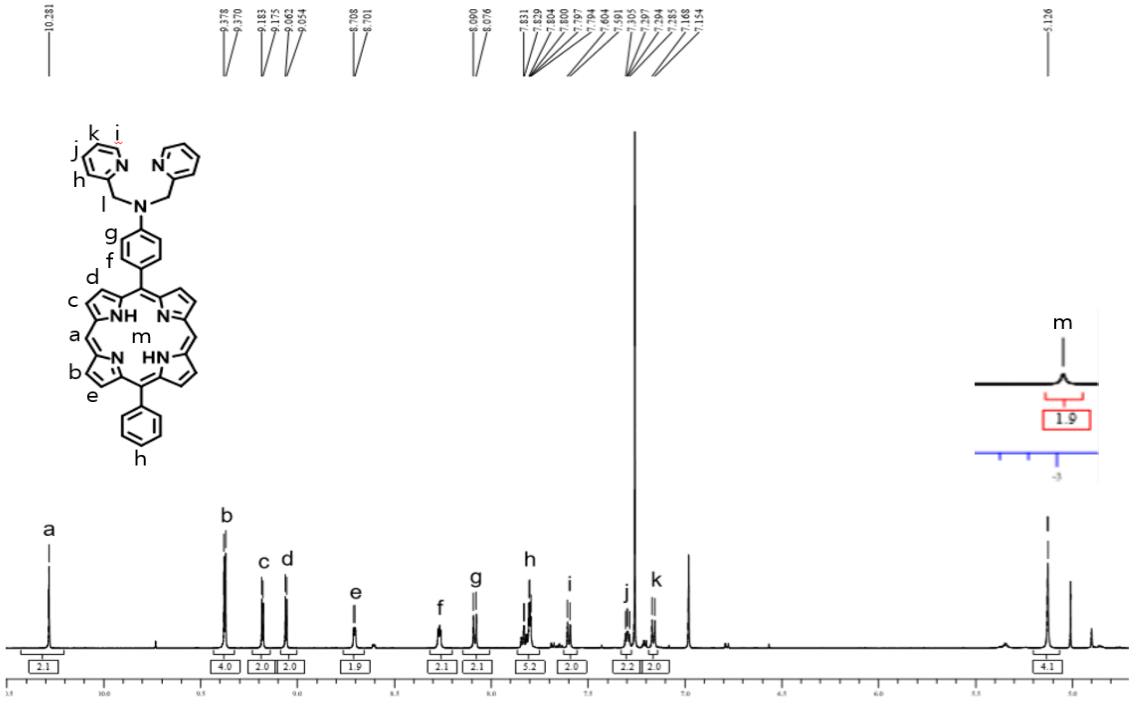


**Figure S3.** $^1$H-NMR spectrum of DPAP.

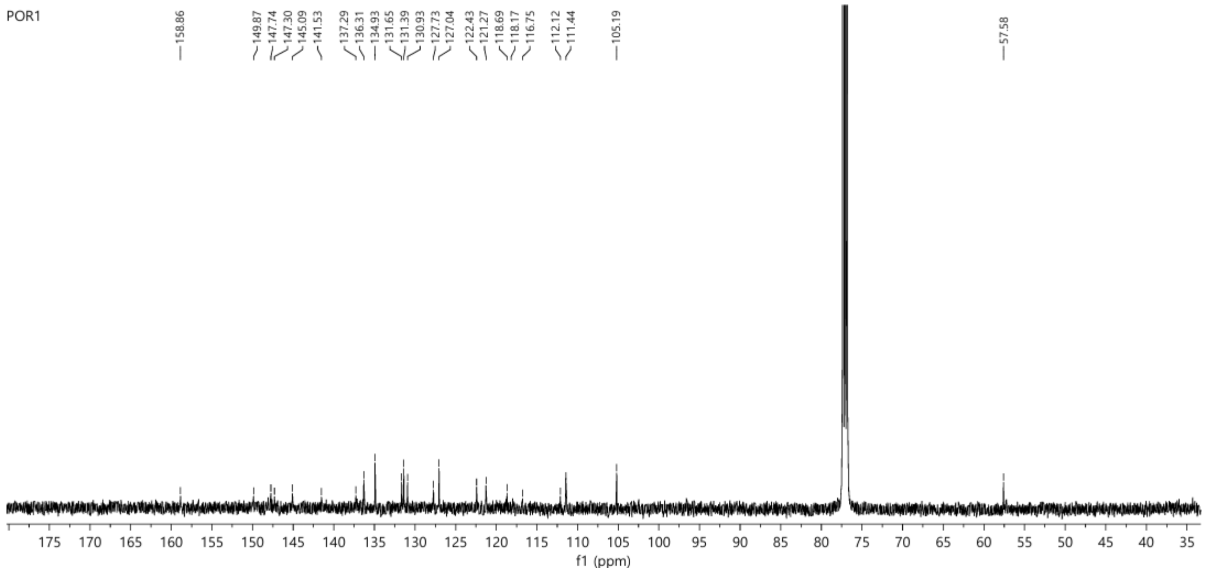

**Figure S4.** $^{13}$C-NMR spectrum of DPAP.

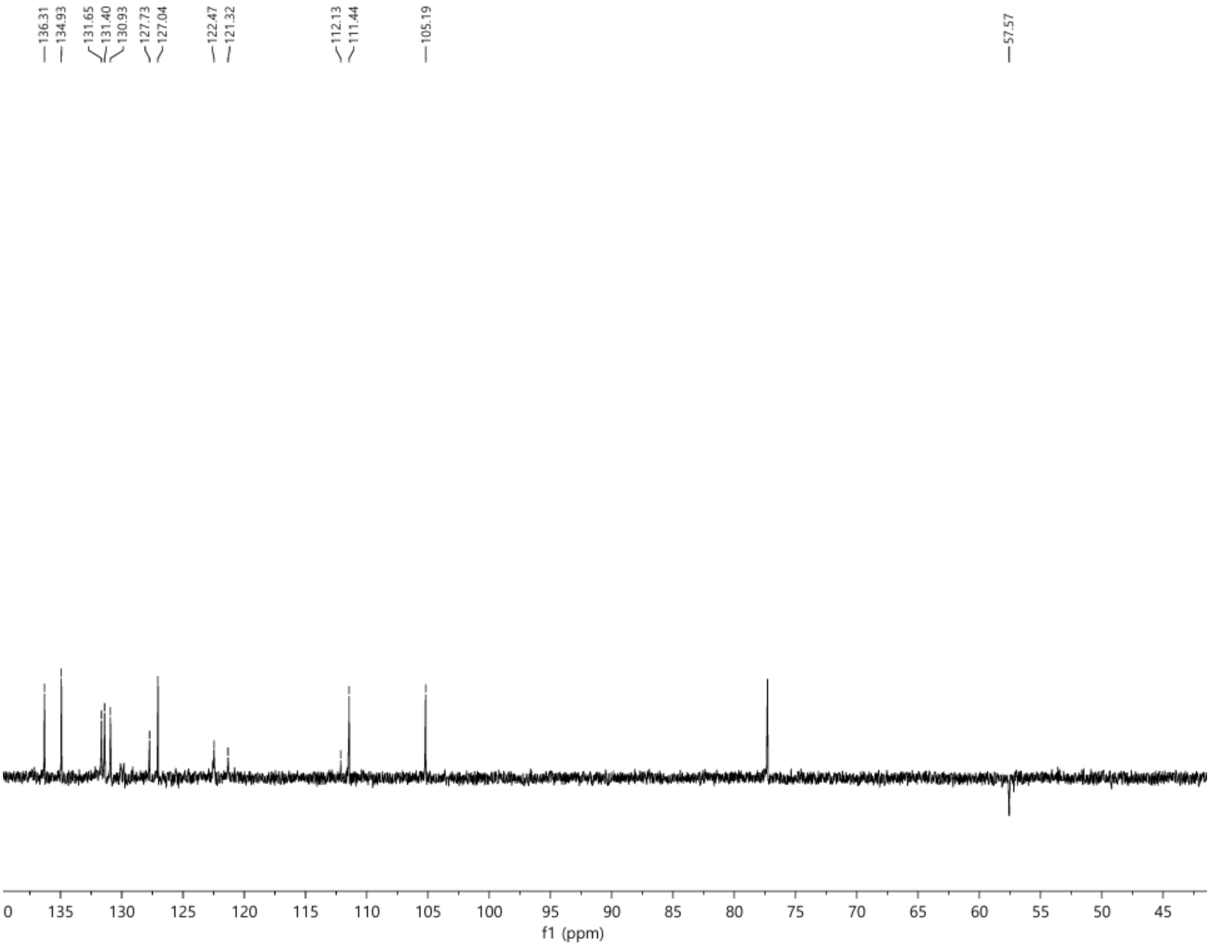


**Figure S5**. DEPT135 spectrum of DPAP

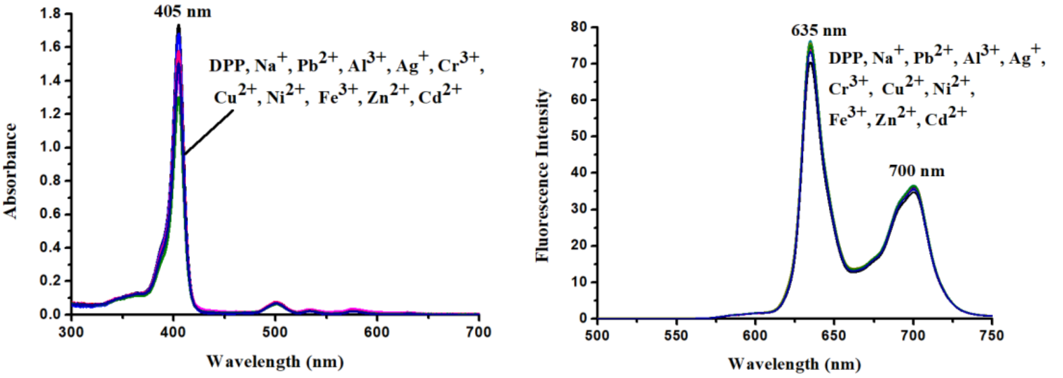


**Figure S6.** (a) Absorption and (b) emission ($\lambda_{ex}$ = 407 nm) spectra of DPP (1.5 × $10^{-5}$M) in the presence of various metal ions (1.5 × $10^{-4}$ M, 10 eq).

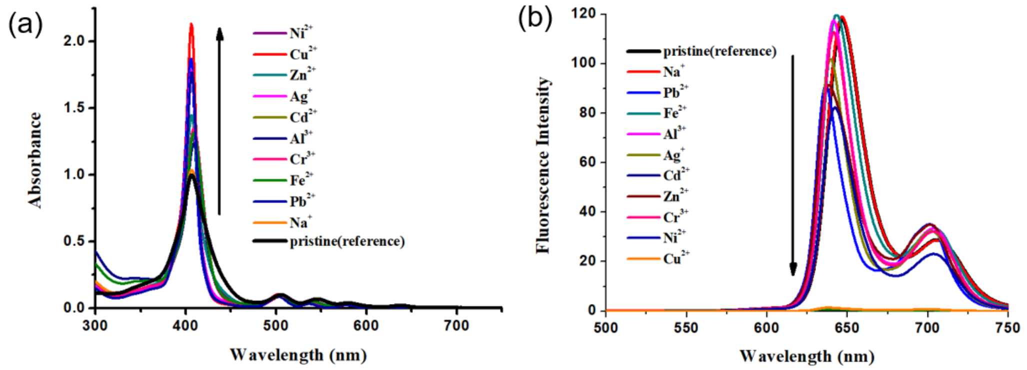


**Figure S7.** Absorption (a) and emission (b) spectra ($\lambda_{ex}$ = 407 nm) of DPAP ($1.5 \times 10^{-5}$ M) in the presence of various metal ions ($1.5 \times 10^{-4}$ M, 10 eq).

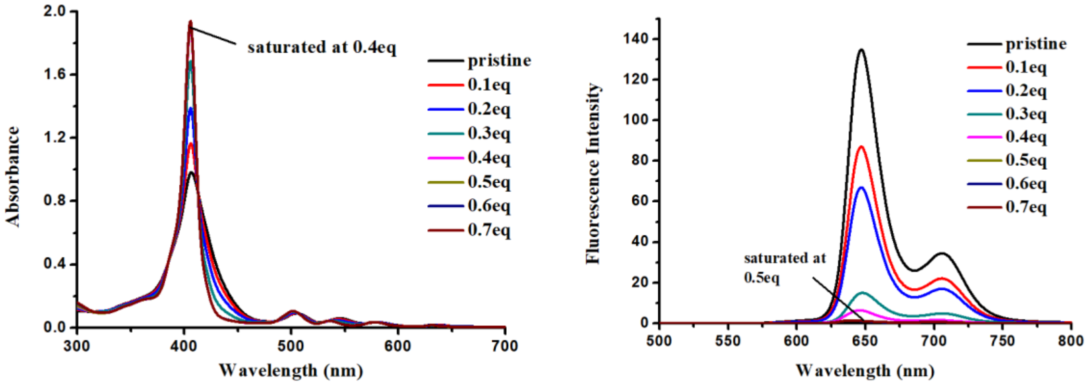


**Figure S8.** Absorption (left) and emission (right) ($\lambda_{ex}$ = 407 nm) spectra of DPAP ($1.5 \times 10^{-5}$ M) with increase in $Ni^{2+}$ concentration.

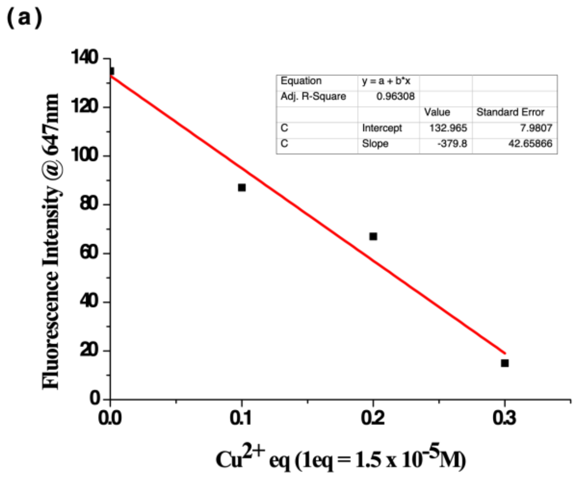


**Figure S9.** Linear plot of the change in fluorescence intensity of DPAP and the determination of the LOD for (a) $Cu^{2+}$ ($k$ = 25.32, LOD = 26.27 nM).

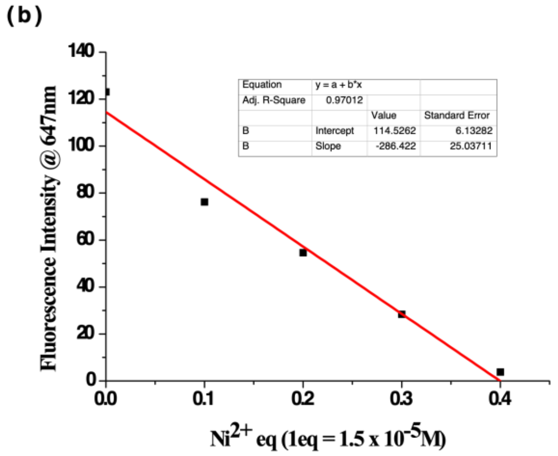


**Figure S10.** Linear plot of change in the fluorescence intensity of DPAP and determination of the LOD for (b) $Ni^{2+}$ ($k$ = 19.095, LOD = 34.84 nM).

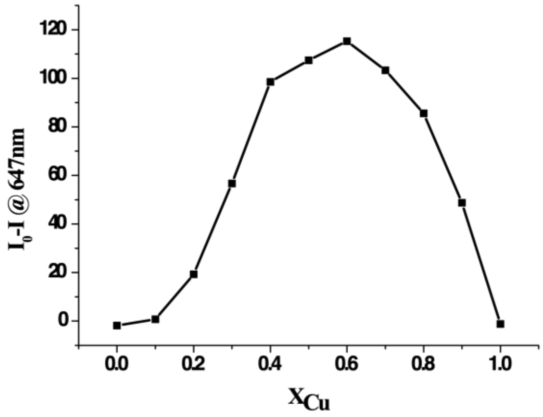


**Figure S11.** Job's plot for the binding stoichiometry of DPAP and $Cu^{2+}$. The *x*-axis is the mole fraction of the cation, and the total molar concentration of the probe and metal ion were kept constant at $1.5 \times 10^{-5}$ M. The *y*-axis is the emission intensity difference ($I_0 - I$) between DPAP and the DPAP-$M^{2+}$ complex at 647 nm.

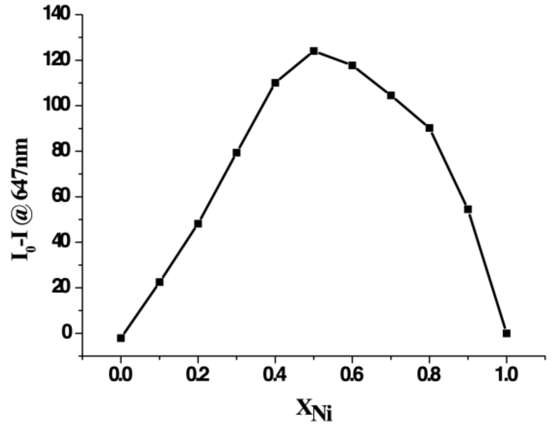


**Figure S12.** Job's plot for the binding stoichiometry of DPAP and $Ni^{2+}$. The *x*-axis is the mole fraction of the cation, and the total molar concentration of the probe and metal ion were kept constant at $1.5 \times 10^{-5}$ M. The *y*-axis is the emission intensity difference ($I_0 - I$) between DPAP and the DPAP-$M^{2+}$ complex at 647 nm.

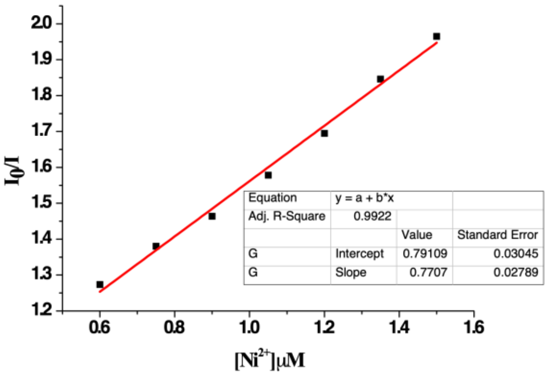


**Figure S13.** Stern–Volmer plot for the quenching emission at 647 nm for the interaction of DPAP with $Ni^{2+}$ ($K_{sv} = 7.71 \times 10^5\ M^{-1}$).

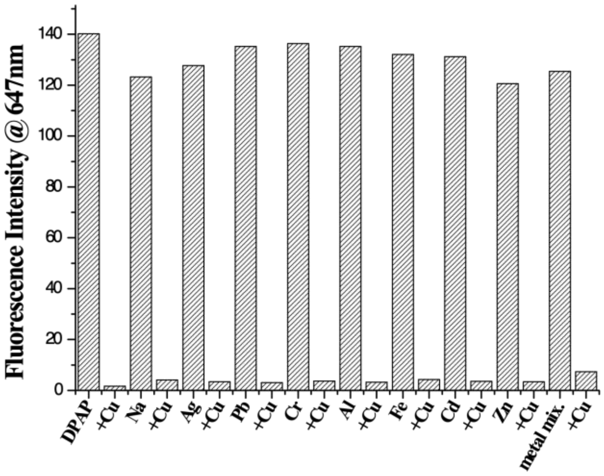


**Figure S14.** Interference effect of each metal ion (10 eq) on $Cu^{2+}$ ions (1 eq) sensing with DPAP ($1.5 \times 10^{-5}$ M). Add interfering ions first and target ion next.

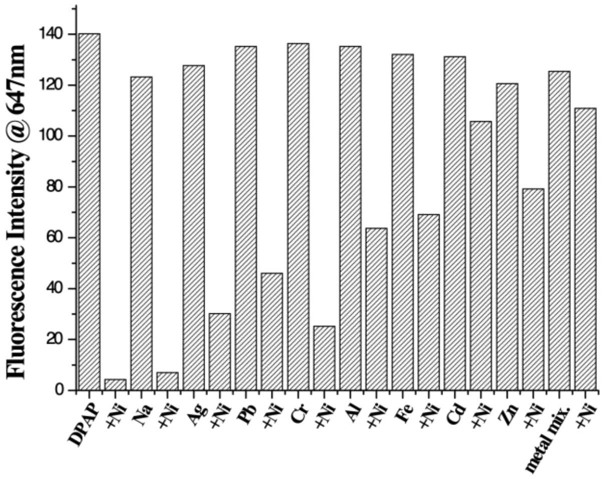


**Figure S15**. Interference effect of each metal ion (10 eq) on $Ni^{2+}$ ions (1 eq) sensing with DPAP ($1.5 \times 10^{-5}$ M). Add interfering ions first and target ion next.

Table 1: Previously reported porphyrin molecules for the detection of $Cu^{2+}$ ions with sensor type, solvent, LOD and response time.

| **Compund Number** | **Sensor Molecule** | **Sensor type** | **Solvent** | **LOD** | **Response time** | **Reference** |
|---|---|---|---|---|---|---|
| Porphyrin 1 | 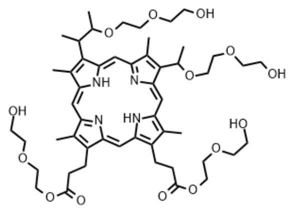 | Turn-off | Aqueous solution | 6.43 nM | 5 min | 33 |
| Porphyrin 2 | 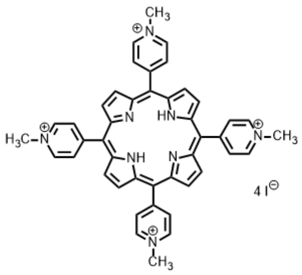 | Turn-off | Aqueous solution | 2.5 µM | 30 min | 34 |
| Porphyrin 3 | 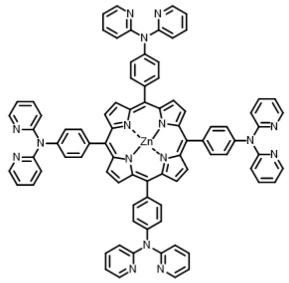 | Turn-off | Chlorofor m/ Methanol mixed solution | 3.3 X $10^{-7}$M | N.A. | 35 |
| Porphyrin 4 | 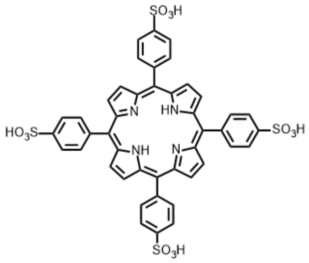 | Turn-off | Aqueous solution | N.A. | N.A. | 36 |
| Porphyrin 5 | 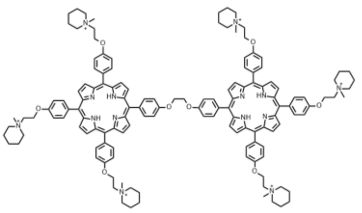 | Turn-off | Aqueous solution | 8.8 X $10^{-9}$M | 10 Min | 37 |
| DPAP | 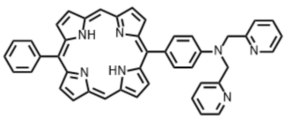 | Turn-off | THF | 26.3 nM | 1 Min | Present Work |
